# Rachel Challice's Vow

**Published:** 1888-02-04

**Authors:** Lina Nevill

**Affiliations:** Author of "A Future on Trust," "Juno," &c.


### RACHEL CHALLICE'S VOW.

By LINA NEVILL, Author of "A Future on Trust," "Juno," &c.

CHAPTER VI.—A WEDDING.

"Oh youth, and love, which is the light of youth,
Why pass ye as the morning?" —*L. E. L.*

THERE was a wedding one November morning in Bedd-gelert Church.

It was neither a large nor grand affair—no ringing of bells, showers of rice, nor bevy of smartly-dressed spectators. The wedding party consisted of the bride, a blue-eyed, fair-haired, peach-complexioned girl; the bridegroom, who was an ordinary specimen of a middle-class young Welshman; and a solitary bridesmaid, who had a pale oval face, dark hair, and grey eyes, and who, as well as the bridegroom, did not seem particularly lively or festive over the affair.

All the gaiety of the ceremony was left to the bride, who, perfectly self-possessed, smiled and blushed, and looked very undeniably pretty.

The clergyman felt cold, and read the service as quickly as possible; for the sexton had not considered it worth while to light the fires for so uninteresting a wedding as that of little Esther Challice. As old Elias Stephens made no secret of his disapproval of the match, little sympathy was felt for David, who was popularly considered to be making a fool of himself, and, like a pig-headed young idiot, recklessly throwing away all his substantial prospects for the gratification of a senseless whim.

The names were duly signed. The clergyman made haste to get into his great coat and comforter, shook hands with the young couple, and gave his stereotyped congratulations, then hurried away to his warm fire at home.

The little bridal party of three followed him more slowly down the churchyard path beneath the old widespreading yew trees. Past the unpretending house, purporting to have been the royal residence of Prince Llewellyn, of Gelert-slaying fame, they went; across the bridge, and so into the main street of the village.

Here Rachel parted with them, and went back to the little cottage on the Ryhd-ddu road; while Mr. and Mrs. David Stephens proceeded up the street, bent on an unpleasant and risky errand—no less bold a proceeding than calling on the obdurate father and imploring his forgiveness, and consequent support towards the expenses of housekeeping.

The wedding had taken place early, for Rachel's convenience; so, although David and Esther had a mile and a-half to walk, Elias had only just finished breakfast, and was looking round for the hat he wore in the mill, when they came in.

"Well," he said, surveying them both with a cold critical stare, "well, and what have you come for?"

"I've brought you my wife, father," replied David, nervously. "I thought you'd like to see her."

"I've seen the young woman many times before; she don't look particularly different. I'm busy this morning, as you've chosen to take holiday, and I must be off to the mill, so I won't ask you to sit down. Are you coming, David, or has your wedding been too much for you?"

"I don't think I'll leave Esther to-day, father, if you can manage without me!"

"I'll do that well enough. How you'll do without *me* is more the question, I imagine. What house have you taken for your wife; and have you found work yet?"

David simply stared at his father, too much surprised for words.

He had never imagined for a moment that Elias would carry his threat into execution. Bluster and angry speeches he had anticipated and was prepared for, but not this cool and firm repudiation.

"I've not taken a house yet," he stammered. "I thought you'd like to have Esther here. She would do for us, and save having in a woman. And—and—I mean to go on helping you in the mill the same as usual; it is only to-day I want a holiday."

"You've made your plans pretty pat, my boy, it seems to me; you and your wife between you, without much consultation besides. I am busy this morning, as I said before, but I'd best make time to lay the state of affairs plainly before you. I won't receive your wife in my house. That's point number one. I've said my say on the subject before, and what I said I'll stick to. As to your working still in the mill, I'll keep you on if you choose, and pay you five-and-twenty shillings a week as I would any other man, and on the same terms—a month's notice on either side, and no favour if you don't do your work properly. Do you agree?"

"I suppose I must," returned David glumly. "You don't give me much choice in the matter."

"I call it cruel and unkind," put in Esther's childish, fretful voice. "Don't accept his offer, David; it's demeaning yourself too much."

"Don't you quarrel with your bread and butter, young woman," observed Elias severely. "I never had much opinion of your sense, and I see now that if David is fool enough to follow your advice in things it will be hard work for you to find the bread, let alone the butter. What's become of Rachel?" he added suddenly. "She has more sense in her little finger than you have in your whole body."

"Oh, Rachel!" replied Esther, tossing her head scornfully. "She is going to London this morning."

"I'm sorry for that," said the old man slowly. "Girls gad about too much in these days. When I was young, my sisters were taught to stay at home and learn to manage the house. It's these excursion trains as does it, making the people unsettled, raising the death rate, and putting money into the parsons' pockets for the funerals. I hope Rachel has not gone in an excursion. I haven't had time yet to see about altering my will, and she'd have been a very deserving object of my bequest."

Elias said this pointedly, looking hard at his son, to show him that his mind was firmly made up on that score; and that the deserving dead of Aberelwyn, and the friends and relatives of such deceased worthies, alone might expect to receive any ultimate benefit from his money.

"I don't think there is any more to be said," observed Elias, firmly settling his hat on his head, an action which caused a little cloud of flour to start out, and frame his face in a white nimbus, like some mediæval saint. "I am going now, and I shall expect to see you at work to-morrow morning, as usual, David."

"Think better of it, Father," exclaimed the younger man. "Remember, I am your only son. Esther, can't you say anything to make him forgive us?"

Esther pouted and looked sulky. Elias's appreciation of Rachel in preference to herself had displeased her, and made her indisposed to be pleasant.

"I am sure he ought to forgive you," she said. "I cannot see, though, what there is to forgive. Why should not you marry like any other young man if you choose. I did not know I was so ugly and disagreeable that people would object to me!" she added, ruffling up her feathers like a little angry bird.

"Your outside is the best part of you," returned Elias coolly. "Leastways for them as prefers the rind to the inside. Now, I always peels the skin pretty deeply off *my* fruit, throws it away, and gives all my thoughts to what's under. David prefers the rind, it seems, so let him have it and welcome. Good morning, now, I've wasted half-an-hour and more in talking to you, and must make up for it. I'll not detain you longer, so if you'll go first I'll follow."

He made steadily for the door as he spoke; and like two dogs detected in some unlawful escapade, David and Esther preceded him, literally driven out of the house by the old man, with their metaphorical tails figuratively tucked between their legs.

Old Stephen went off to the mill, a grim smile on his hard features; and now and again, as he watched the fast revolving machinery and the ceaseless whirl of fine white dust, a low, self-satisfied chuckle broke from him. No one appreciated victory, or rejoiced more in the total rout of his enemies, than did this despotic old man.

David and Esther stood in the road outside the house—that neat, trim, yellow-washed, purple-slated house in which they had fondly designed to live—surveying each other disconsolately, and looking as unlike a gay young bride and bridegroom of a few hours' standing as it was possible for them to appear.

"What shall we do?" asked David at length.

"Can't we go home?"

"I don't see how we can," responded David gloomily. "Rachel was only going back to pick up her things on the way to Ryhd-ddu station; and the Howels were coming in at ten o'clock. They would scarcely care to find you and me there. They have bought all the things, too, as they stand, and would have every bit as much right to turn us out as father has just done, and more. Once in a day is quite enough of *that* performance. I haven't much relish for going through a repetition of the game."

Esther, not knowing what else to do, took refuge in her usual habit when annoyed or put out—she began to cry.

"Stop that. Don't be silly, Esther," said David impatiently, in very unloverlike tones. The sight of Esther's tears was the final straw to his perplexities. And fretful, uncalled-for tears at any time exasperated him—as they do most men—and made him feel vicious.

"I do call it hard to have nowhere to go to on my wedding morning. Rachel should have managed better, and not left us without breakfast or a chair to sit down on," whined Esther, glad to have a fling at her innocent, long-suffering, scapegoat sister, in revenge for Elias's preference of her. "She should have stayed on another week at least, and made things comfortable for us. But then Rachel always was hard and selfish. Poor dear Granny always used to say so."

"I don't see how Rachel is to blame for this," replied David, a little too warmly for Esther's peace of mind, ruffled as it had been before.

"Of course you'll stand up for her," retorted his wife. You always were so fond of her; I only wonder you didn't go and marry her!"

"Perhaps I might, if I hadn't married you," he replied, dryly. Then, with a touch of his father's cynicism, he continued—"It is no use crying over spilt milk now. You have just taken me for better or worse, my dear. It is for worse at present, I am sorry to say; so make the best you can of a bad job, and come and see if we can get old Mrs. Powell to put us up for a week or two till we can look round us, and get into a house of our own."

Esther dried her tears, fearful of making her eyelids red if she cried longer. Besides, as they had no effect upon David it was sheer waste to go on shedding them.

She refused the aid of the arm he awkwardly tendered her —under the impression that brides and bridegrooms always walked about arm-in-arm on their wedding-day—and marched sulkily along by his side; beginning seriously to doubt whether marriage, on which she had so firmly set her heart, was after all so blissful a condition of life as she had, from a distance, fondly believed it to be.

*(To be continued.)*

